# A modified theory of planned behavioral: A case of tourist intention to visit a destination post pandemic Covid-19 in Indonesia

**DOI:** 10.1016/j.heliyon.2021.e08230

**Published:** 2021-10-21

**Authors:** Pahrudin Pahrudin, Chien-Ting Chen, Li-Wei Liu

**Affiliations:** a Department of Business Administration, Chaoyang University of Technology, Taiwan; b Department of Physical Education, Chung Yuan Christian University, Taiwan; c Department of Leisure and Service Management, Chaoyang University of Technology, Taiwan; d Faculty of Social Science and Economic, Universitas Hamzanwadi, Lombok, Indonesia

**Keywords:** Post Covid-19, TPB, Non-pharmaceutical intervention, Health consciousness, Intention to visit

## Abstract

The Pandemic covid-19 spread globally has been given impact in the tourism industry, especially in the tourism destination. This study investigated to build the concept and theoretical framework that explains the decision of local tourist intention to visit a local destination in Indonesia post-pandemic covid-19. This study was considered the perception of Covid-19, non-pharmaceutical intervention and health consciousness by implying of Theory of Planned Behavior constructs. This study, health consciousness is the moderator variable to predict the decision of tourist to visit a destination. Structural Equation Model-Partial Least Square (SEM-PLS) was used to analyze the construct of study. The model found that the Theory of Planned Behavior was successfully broadened in making the decision of tourist to visit a destination post-covid-19 with considering non-pharmaceutical intervention and health consciousness. The results showed that generally the constructs of Theory Planned Behavior are significantly impacted in intention to visit a local destination in Indonesia, except Hypothesis of subjective norm and intention to visit was rejected. The variable health consciousness through intention to visit also was rejected. The framework also used moderating variable health consciousness between subjective norm and intention to visit was rejected. This study was given insight an issue of covid-19 in the tourism sector, and the implication was providing government, stakeholders, tourism marketers and policy-making with considering non-pharmaceutical and health consciousness during and post-pandemic covid-19.

## 1 Introduction

Tourism is one of many sectors affected by the disaster, either natural disaster or pandemic/epidemic and making an impact of tourist intention to visit a destination. The illness or pandemic is one categories of natural disaster that given influence to tourist behavioral. A natural disaster such as Earthquake, Tsunami, Eruption, Floods, spread illnesses or pandemic can give a significant impact on the tourism sector. A natural disaster could give impact both international and domestic tourist to intention to visit such as natural hazard, crisis 9/11, SARS pandemic in Asian continental and destruction appeared by tsunami hazard (Page et al., 2006). The epidemic of Avian Flu was declined the international visitors around more than or at least twelve million in Asia Pacific regions (Wilder-Smith, 2006). In another side, SARS pandemic has made impact their job lost approximately three million people and generally countries of Asia such as China, Hongkong, Vietnam and Singapore (WTTC, 2003).

Coronavirus or Covid-19 the first time founded in central China, Wuhan in late December 2019. SARS has emerged in 2003, has similarity with Covid-19 because of mobile illness health that extremely infection between humans or transmission human to human (Wen et al., 2020). Pandemic covid-19 has been given impact in tourism sector in the world include Indonesia country since March 2020. It seems clear that COVID-19 has reduced visitor in the tourist sector since its pandemic, which has greatly impacted in reducing the income of tourism industry as a whole (Jhonson and Boone, 2020; UNWTO, 2020a) and has changed travel behavior of tourist (Vos, 2020).

The covid-19 was giving significant impact to whole life such as economic sectors, health sector, environment sector, social and culture sector, education sector, and tourism sector. Covid-19 was giving significant impact globally in the economic sector and tourism sector: international tourist arrivals are predicted decrease in tourism sectors down to 78% and lost income around US$ 1.2 trillion, works were lost around 120 million the greatest decrease in this era (UNWTO, 2020b). The tourism sector is one of the sectors being the most critical role in global employer and the give contribution to GDP for some countries in the world (UNWTO, 2020b).

However, few tourists have an awareness of how health an urgent when they are planned to travel during pandemic covid-19. Understanding their behavior is essential to help stakeholders, government, hotel services, tourism marketers in the handling of crisis pandemic covid-19 more effectively and efficiently because tourist can use non-pharmaceutical intervention to help them safe when travelling to a destination. This study classified the perceived risk in health for covid-19 that appeared in the world as a barrier which could give an impact in health risk to tourist from visiting the destination.

For example, if those who are planning to visit destination perceive risks from covid-19, such as spread the illness, fever, cough, they could cancel the travelling because of level perceived safety (Reisinger and Mavondo, 2005a). In the pandemic era, tourist could reduce perceived risk during visit tourism destination with considering personal non-pharmaceutical interventions from covid-19 infection while travelling. Personal non-pharmaceutical interventions are of the best way to avoid spread the illness while travelling include the knowledge of pandemic, health life practice, social distancing and checking personal health pre and post-trip (Nicoll, 2006).

Many researchers in the field of the tourism sector, psychology of social and behavior of consumer believe that perceived risk elements that caused to specific intention to make a decision or desirability to visit a destination (Han, 2015; Vos, 2020; Wu et al., 2017a, Wu et al., 2017b). The concept and framework in this study are adopted form Theory Planned Behavior and health perspective to test or examine their planned and intention to visit a destination according to pandemic covid-19 (Ajzen, 1991; Chen and Tung, 2014; Juschten et al., 2019; Lee et al., 2012; Li et al., 2020; Zeng et al., 2021). The theory of TPB has strong to know more about sociopsychological theory to foresaw the behavior and understanding the decision of tourist in intention to visit a destination (Ahmad et al., 2020; Eom and Han, 2019; Ulker-demirel & Ciftci, 2020).

Tourist behavior is the principal problem in the tourism sector, especially when a risk appeared to visit a destination by tourist during the covid-19 pandemic. In the previous study, there are some studies about theory of planned behavior (TPB) generally applied in the tourism sector to know how tourist intention to visit a destination (Jamal and Budke, 2020). However, some of the studies in the tourism sector related to covid-19 and theory of planned behavioral focus on these theories have limitedness. Therefore, this study was expanded with combining among TPB Theory, covid-19 perception, non-pharmaceutical intervention and health consciousness that related to tourist decision in intention to visit a local destination in post-pandemic covid-19.

This study is embedded as a part of theory planned behavioral on tourism sector related to covid-19 perception and non-pharmaceutical intervention (NPI) that give impact in intention to visit a destination during the pandemic and post covid-19 in context of Indonesian local tourist. This study gives knowledge contributions about intention to visit a destination using Theory Planned Behavioral in the post-pandemic covid-19 era and also the current study was conducted in a part of the contribution to the theoretical improvement in tourism sectors related to health field during covid-19 pandemic thus enhancing understanding tourist behavior that may impact intention to visit. This paper expected to provide insight for tourism industry to use the Theory Planned Behavior framework related to covid-19 and health perspective as part to support policymakers to develop prompt and action policy in local tourism destination in Indonesia.

This study investigated to build the concept and theoretical framework that explained the decision of Indonesian local tourist to intention to visit a destination in post-pandemic covid-19 with considering health indicators by implying of TPB. This present study contributes to the enhancing research on the influence of the travelers in intention to visit a local destination post-outbreak covid-19, integrated with theory of planned behavior (attitude, subjective norm, perceive behavior control) and health sectors. In addition, we investigated how health sectors such as health consciousness and non-pharmaceutical intervention could influence the study framework. This value enriches the relation and consequence of the visitor behavior influence visit intention a local destination in the post-outbreak covid-19 period. The aims this study are: 1) to expand the Theory of Planned Behavior in intention to visit a destination post-pandemic, 2) discover of variable perception of covid-19 in intention to visit a destination in post-pandemic covid-19, 3) to explore the role of moderating variable to research construct and proposed conceptual framework of research.

## 2 Literature review and hypothesis development

### 2.1 Tourism sector and Covid-19

The previous research on tourism related to epidemic disaster has urgently discussed on examining the recovery pattern by (Cheer, 2020), the effect of diseases on tourist arrival (Shi et al., 2020), and research on how the pandemic can change the tourist behavior and preferences (Wen et al., 2020) and examining impact on economic sector. However, the decision of tourist to make decision to visit a destination during and post pandemic is still restricted (Sánchez-Cañizares et al., 2021). Therefore, the researcher from (Chuo, 2014) examined the customers behavior related to self-protection in dining industry and from (Lee et al., 2012) related to health protection with the impact of non-pharmaceutical intervention on visit intention of international tourist to visit.

However, Indonesia is one of the countries that has been impacted by covid-19. As of September 15, 2021, there were 4.174.216 confirmed cases and 139.415 deaths (The Indonesian COVID-19 Task Force, 2021). In travel industry, based on Indonesia Travel Agent Association announced that the decreased in sales of up to 90% with potential loss around US$245 million due to cancellations (The Jakarta Post, 2021). International flight arrived and depart also indicated a drop of more than 95% compare to 2019 (Wibawa, 2020). In Indonesia's hotel industry also has been impacted by covid-19 with hotel occupancy was only 19.7% June 2020 compared with 52.3% for same month last year (The Jakarta Post, 2021). All these data are reflected in changing whole sectors such tourism and hospitality industry and also have affected the behavior of local tourist visit intention because risk perceived from the pandemic covid-19.

Therefore, using the theory of planned behavior is useful to predict tourist behavior to visit a destination during or post pandemic covid-19. The theory of TPB has been applied in several sectors but has not been applied in pandemic context except variable of intention in medical aspect (Agarwal, 2014; Zhang et al., 2020); study from (Lee et al., 2012) uses TPB theory in intention to visit during health crisis (H1N1). However, since the pandemic covid-19 spread over the world, the theory planned behavior has used this theory to the same ends (Bae and Chang, 2021) and analyze behavioral intention towards ‘untact tourism’ in South Korea (Li et al., 2021); focus on post-pandemic intention to travel in China; and (Wang et al., 2021) examine Chinese residents' intention to use hotels after Covid-19.

In this research, we conducted research with applying TPB constructs, health perspective and pandemic situation (covid-19). Theory of planned behavior constructs are the main frame and mixing with the variable of health perspective such as health consciousness, non-pharmaceutical intervention, change of intention related to health, and pandemic situation (perception of covid-19) in intention to visit a destination post pandemic-covid 19. Therefore, it is important to understand the tourist behavior whether the post pandemic covid-19 tourism can be expected follow the pattern recovery rapidly similar after the SARS and MERS epidemics, and to evaluate the factors that influence the tourists’ in making the decision to visit a destination post pandemic covid-19. The next section explained the outline factors that are important related to tourist visit behavior post-pandemic covid-19.

### 2.2 Theory of planned behavior and intention to visit

The Theory of Planned Behavior explains about the behavior of individual related to intention. TPB has been used in several fields such as marketing, health sector, and tourism sector to know about human or individual behavior. TPB is one of the theories used in sociopsychology theories about the predict human intention/behavior. This theory was built based on theory reasoned action (TRA) by (Ajzen and Fishbein, 1977). The primary variable of Theory reasoned action is an attitude and subjective norm that give an impact on behavior intention, which is an impact on actual behavior. Therefore, Attitude, Subjective Norm, and Perceive Behavioral Control are the main subjects to explain behavioral intention in the theory of TPB (Ajzen, 2001; Ajzen and Kruglanski, 2019).

Intention process is divided into two main points (1) attitude towards behavior and (2) subjective norm, on the other side perceive behavior control is the main factors of non-volitional processes (Ajzen, 1991; Perugini and Bagozzi, 2001) Behavior intention is the main proximal of absolute behavior/action in the TPB framework (Eom and Han, 2019; Guerin and Toland, 2020), and intention is the part of attitude toward behavior, Subjective Norm and Perceive Behavior Control in TPB framework (Guggenheim et al., 2020). In other words, volitional and non-volitional constructs an individual intention (Guerin and Toland, 2020; Song et al., 2017). Theory of TPB was built from TRA (Ajzen, 1991, 2001). However, TRA is the dimension of willingness or readiness to predict human behavior (Ajzen, 2001; Ajzen and Kruglanski, 2019; Guggenheim et al., 2020).

Tourist intention or decision to visit a tourism destination is influenced by attitude and behavior (Guggenheim et al., 2020; Han and Hyun, 2017; Kim and Hwang, 2020). It means that concept of attitude indicated general personal assessment about the particular behavior that evaluated positively or negatively (Ajzen, 2001; Perugini and Bagozzi, 2001). Subjective norms refer to a critical of tourist to do something that refers to behavior intention (Song et al., 2017; Wu et al., 2017a, Wu et al., 2017b). This means that Subjective Norms are a personal concept in social context to do an action or do not act in the context of behavior (Ajzen and Kruglanski, 2019; Guerin and Toland, 2020).

The other factors that give impact in determining of tourist intention are perceived behavioral control (Kim and Hwang, 2020). This factor as a non-volitional factor that refers to personal perception in their ability toward an activity related to behavior (Perugini and Bagozzi, 2001). The theory of TPB consists of Attitude, Subjective Norms, Perceive Behavioral Control and Intention have a positive relationship and has been tested and extended in tourism, and consumer behavior studies (Hwang et al., 2020; Kim and Hwang, 2020; Wu et al., 2017a, Wu et al., 2017b). study from (Ajzen, 1991) revealed that concept of TPB has empirically-supported, in which positive attitude towards the action, perceive social pressure and perceive the ability to take action to have a positive effect to customer's behavioral intention.

The pandemic covid-19 can make influence the tourist travel behavior and try to avoid the travel because of the pandemic risk (Gupta et al., 2021), received the risk due to outbreak (Li et al., 2020), and change the intention or psyche of tourist (Kock et al., 2020). In this session, it can be concluded that the tourist behavior could be change in perception about the perceived risk perception and types of the destination (Li et al., 2020) and kinds of the tourism (Zeng et al., 2005). In case of post pandemic, the tourist would be try to visit a safer destination with fewer risk spread of illness and try to avoid the high risk (Gössling et al., 2021). The pandemic covid-19 has influenced the behavioral and level psychological of human such as fear, panic and suspicion (Zheng et al., 2021).

Theory of planned behavior is explained about the behavior related to intention to visit a destination during the pandemic covid-19. This theory has been applied in the different area (Agarwal, 2014; Zhang et al., 2020), it has not yet been applied in the post pandemic context except model intention variable on medical field from (Lee et al., 2012) applied in tourism sector during the risk H1N1 to discuss potential tourist's visit intention a destination.

Using some kinds of literature, three antecedent Theory Planned Behavioral (TPB) variables for attitude, Subjective Norm and Perceive Behavioral Control were hypothesized to have a significant impact on the intention to visit a destination post covid-19. These were:

Hypothesis 1 Attitude has a significant impact on intention to visit a destination post-covid-19;

Hypothesis 2 Subjective norm has a significant impact on intention to visit a destination post-covid-19,

Hypothesis 3 Perceive behavioral control has a significant impact on intention to visit a destination post-covid-19.

### 2.3 Perception of Covid-19, non-pharmaceutical intervention and intention to visit

The recent pandemic covid-19 is one of the infectious diseases since the new era began. There is some pandemics disease such as SARS in 2003, the virus influenza H1N1 in 2009, the Ebola disease in 2014 and MERS during 2015 (Huang et al., 2020). The pandemic made an impact in several sectors, including tourism sectors. People perception about the disease when confronted with infectious such as diffusion, dead/mortality, affected case by disease and fever can make influence the decisions tourist to visit a destination (Huang et al., 2020).

Some researchers said that covid-19 came from bats and transmission to human. The researchers explained that the spread of illness moving into humans through the virus. Covid-19 have found from Wuhan in China and then spread into the whole of the world and WHO declared that virus covid-19 is pandemic phenomena, which means that virus has spread globally (Medical News Today, 2020).

Transmission covid-19 moved to human through body organ such mouth or nose when the people cough or breath out (Wisconsin Department of Public Instruction, 2020). Transmission covid-19 moved to human through body organ such mouth or nose when the people cough or breath out. The person who infected covid-19 when the person has touched the object or surface then touch body organs such as nose, eyes or mouth. The human can get covid-19 illness through person transmission when coughing or breath out. Hence, it is essential to keep distancing from the group gathering (United Nation, 2020).

World Health Organization announced that pandemic virus H1NI 2009 significantly enhance to six-level phase because the amount person of infected the virus increased globally (Lee et al., 2012). St. Michael's Hospital said that transmission of epidemic very fast via air transportation such airplane because the airline can move the passenger from one place to another (Khan et al., 2009). In case of covid-19, Since early February 2020, more than fifty airline companies cancelled aviation from and to China and some countries in the world such as Italy, Australia, Russia, and the USA, and have also travel warning issued by the government (Chinazzi et al., 2020).

Consumer perceptions (tourist perception of covid-19) are information and response about their understanding of knowledge, procedure, behavior and matter (Lee et al., 2012). During pandemic crises gave huge impact and negative perception in the tourism industry, hospitality and destination. Some cases such epidemic Ebola 2014, although Africa was not affected by Ebola but gave impact for those who will travel because of the evidence and adverse insight (Maphanga and Henama, 2019; Novelli et al., 2018). Therefore, when the global pandemic needs quarantine and lockdown, the consumer or tourist perception of the disease and its implication in the new normal in tourism and hospitality requires further discussion.

Health protection strategies (e.g. social distancing, the campaign of stay of home or work from home, self-quarantine, closing travel or travel bans, keep distancing) have quieted in global visiting in tourism and leisure. The tourism sector is one of the categories of the industry that susceptible and become resilient from several risks such as virus or pandemic, terrorism, earthquake, Ebola, SARS and Zika (Novelli et al., 2018). The main feature of this pandemic is that it has had a significant global impact and has led to recessions and depressions around the world (Sigala, 2020).

NPI is one of the interventions that control measures and control system to protect personally from disease (Raude and Setbon, 2009). There are some ways for Non-pharmaceutical intervention, including physical distancing or social distancing, individually quarantine, health control regularly, and cleanliness surveillance (Oshitani, 2006). Keep distancing during a pandemic can avoid the spread the illness of disease. Social distancing means that keep distancing with others. There are some steps can be applied in social distancing by work from home, closing the public places (school, market and etc.), avoid mass gathering, reducing access public transportation, and diminish contact by personally. Hygiene behavior by washing hands with soap in water, wear the mask, using techniques in coughing are the best way to restrict disease spread (Finkelstein et al., 2009). Since the pandemic era, components NPI such as self-isolation, quarantine and contact tracing were important things to avoid the disease (Omy, 2006).

The Non-Pharmaceutical intervention is one of the action methods like closing schools or public area, banning public gatherings, keep distancing, self and group quarantining ill patients. Several countries in the world are used thermal screening to detect the passenger who has a high level of temperatures in the gate. The passenger will be detention phase to an alleviation phase when the illness appeared in the groups. For example, several flights banned in several countries such as the flight from Argentina to Mexico, flight from the United States of America to Mexico country Border, and country of New Zealand filtered the passenger (Department of Health and Human Services., 2009). Virus H1N1 outbreak in 2009 explained the complexities of implementing NPI controls globally. Indeed, during March and April 2008, the number of tourists arrived around 2.35 million that use airplane from Mexico, with the same design predicted in the same month when the virus H1N1 2009 appeared (Khan et al., 2009).

Some of the circumstances that passengers must understand to avoid travel that has the impact of contracting the virus influenza on airlines (St. Michael's Hospital, 2009). The firstly, incubation time of the disease is greater than usual. Second, the passenger who has experience in symptoms could not recognize their condition because of travel regulations. Third, the passengers of airplane generally are not available for rapid screening and diagnostic technique for infectious disease. Hence, passive management at national boundary-less effective because the difficult to stopping the passenger in arrival or departure that related with infectious diseases such as influenza and their near symmetry between pairs of international airports means sharing the risks of exports and imports of sick passengers. In addition, preventing a pandemic for a few weeks or more than as usual would need 99% because of the effect of control in border control and internal trip examine (Ferguson et al., 2006).

Virus outbreaks spread rapidly, and the authorities concerned have the right to identify the new virus—for example, the epidemic H1N1 2009 from Mexico with a rapid and widespread spread. Therefore, disease recognition, surveillance and person-to-person disease prevention attitudes on planes and at airports require the cooperation of all countries of the world (St. Michael's Hospital, 2009). The fast circumstance mobility complexity; antivirus and vaccine efficacy and availability; and non-pharmaceutical intervention such travel postponed, which means stopping the pandemic is an increasing global challenge. This outbreak gives impact to tourism sector significantly because of the tourist behavior increased risk perception and discomfort due to information, preparedness, and treatment of disease (Wu et al., 2010). So, when an outbreak vigilant has appeared, intention to visit a local destination, their perception of covid-19, health and personal NPI protective measure for the disease need further investigation.

The obtaining information and knowledge about the disease and the pandemic, tourist can improve their personal health while travelling such as life hygiene, social or physical distancing, wear the mask and etc. The study proposed that perception of covid-19, non-pharmaceutical intervention and intention to visit as follows.

Hypothesis 4 Perception of covid-19 has a significant impact toward non-pharmaceutical interventions.

Hypothesis 5 Non-pharmaceutical intervention has a significant impact toward intention to visit.

### 2.4 Health consciousness as a moderator variable and intention to visit

Most of the theories said that health consciousness related to motivating of preventing the disease or the primary behavior from improving the health (Newsom et al., 2005). In other perspectives, Health consciousness refers to the level of preparedness to do healthy, which built widely of the person about his own health (Ophuis, 1989; Schifferstein and Oude Ophuis, 1998). Health consciousness of consumers refer to concern about their health and motivating their health and quality of life with engaging healthy behavior and being self-consciousness about health. Health consciousness is aware and care about healthy to increase or keep a quality of life with healthy action with caring about their own health (Kraft and Goodell, 1993; Newsom et al., 2005). Some studies about the consciousness of health revealed that the consumer's attention about issues of health food (Fagerli and Wandel, 1999; Rozin et al., 1999), hence, the consumer are considered about the health and quality of food in the decision of food buying (Magnusson et al., 2001; Wandel and Bugge, 1997).

Based on the theory of self-consciousness, self-awareness can predict the attitude and behavior with expands to health consciousness and health behavior (Gould, 1990). Hence, there is an important thing that stability between health consciousness and health behavior. Health consciousness refers to knowledge of people about the health themselves, take attention of individual about health concern, and make sure about their personal health (Piko and Keresztes, 2006). During pandemic covid-19, the important of tourist deeply knew about health while travelling, and they could adopt health attitude and norm to protect their health. In the tourism sector, health consciousness is very important to avoid the spread the illness of covid-19 of tourist during travelling in the future. There are some studies said that the people with high level health consciousness tend to gain healthier lifestyle (Gould, 1990; Jayanti and Burns, 1998). In other some studies said that the role of health consciousness in health behavior such as buying healthy food and seeking medical service (Justina et al., 2017; Mai and Hoffmann, 2017). However, as a moderator variable, health consciousness had not significant impact through the subject norm and intention to visit.

Therefore, apart from health awareness, how tourist attempt to express their identity related to health consciousness arena is studied further here. In addition, if a tourist has a higher level of health an awareness than more willing to engage in activities that are directly related to health such to avoid the intention to visit a destination because of illness covid-19. Conversely, tourists who have a low level of health awareness will find it difficult to avoid a visit a destination during pandemic covid-19. Therefore, it would be helpful to explore whether these two constructs have a moderating effect on attitude and subjective norm toward intention to visit a destination post-pandemic covid-19. Based on the literature above, the subsequent hypotheses are proposed:

Hypothesis 6 Health consciousness has significant impact toward intention to visit.

Hypothesis 6a Health consciousness as a moderator variable has a significant impact toward intention to visit through attitude.

Hypothesis 6b Health consciousness a moderator variable has a significant impact toward intention to visit through the subjective norm.

### 2.5 Health-related to visit intention a destination

This study was applied to TPB based on the perception of tourist in health perception and visit a tourism destination post-covid-19. Health perception related to visiting a destination during a pandemic is part of risk because tourist can get the illness of virus. In many studies, tourist behavior during the pandemic or epidemic, e.g. Ebola disaster, SARS and other outbreak is part of theory risk (Cahyanto et al., 2016). Based on the theory of risk, tourist will be considering the risk and prevent the negative experience. In the other hand, high perceive of health risk. Based on the risk theory, tourists tried to avoid the negative experiences and always maximize their satisfaction. In other word that high risk perceive gives impact on behavior lower buying (Lim, 2003). Many studies said that tourist travel intention directly influenced to risk perceived (Al-Ansi et al., 2019; Olya & Al-ansi, 2018; Reisinger and Mavondo, 2005b). In marketing studies, the risk was found by (Bauer, 1960), and said that consumer behavior contains both risk and uncertainty caused by their action and some unpleasant.

In the first study in relating to risk studies and uncertainty as a two-component that identic as a research construct (Shimp and Bearden, 1982), in which risk is identified the feeling of the person about the result of the decision will be given positive and profitable. Nowadays, many studies that related of risk to marketing and tourism sector (Mohseni et al., 2018; Tseng and Wang, 2016; Wu and Cheng, 2018).

The second study tries to compare between uncertainty and risk. In this study perspective, the risk can be measured in ratio of events to the total possible result (Robert & Grønhaug, 1993), while uncertainty is defined as a circumstance in which the outcome could be anything that is not prompted. The researchers (Robert & Grønhaug, 1993) have found that the definition of perceived risk is possible in the future about the loss the incurred when the decisions have been made. In other words, risk perception in uncertainty and perceived riks in context of marketing and tourism concepts have a different definition.

Risk perceived is anticipation of possible of disappearance where the advantage is attached to the possible consequences (Dowling and Staelin, 1994). as the result of the risk perceived, the people see the comparison of kinds of risk and related on the result. However, based on (Becker and Knudsen, 2005), uncertainty is defined as the probability of disappearance that can be attached to a possible result. Moreover, since the perceived risk is defined a type of possible of loss, researchers (Dholakia, 2001) said that there are kinds of potential risks in term of performance, financial, psychological, social, and finally the time risk (loss) aspect.

On the other hand, if a global pandemic occurs, the behaviour of tourists in the tourism sector is influenced by several main factors such as home income, perceptions of health risks, and consumption measures that are reformed due to pandemic constraint (Lee and Chen, 2011) in the tourism sector, all the factors indicated are important in increasing the tourist behaviour related to intention to visit a destination. Morever, the covid-19 disaster is a significant part of the risk, especially are parts of perceived risk especially health risk, and as a result of the relationship between the perception of pandemic risk and tourist confident. The health predictor can change of intention to visit a destination in post-covid-19. Based on the literature framework above, the subsequent Hypothesis is proposed:

Hypothesis 7 Intention to visit has a significant impact toward health related change of intention.

In the bellow is the proposed theorical model of the study.

## 3 Method

**Figure 1:**
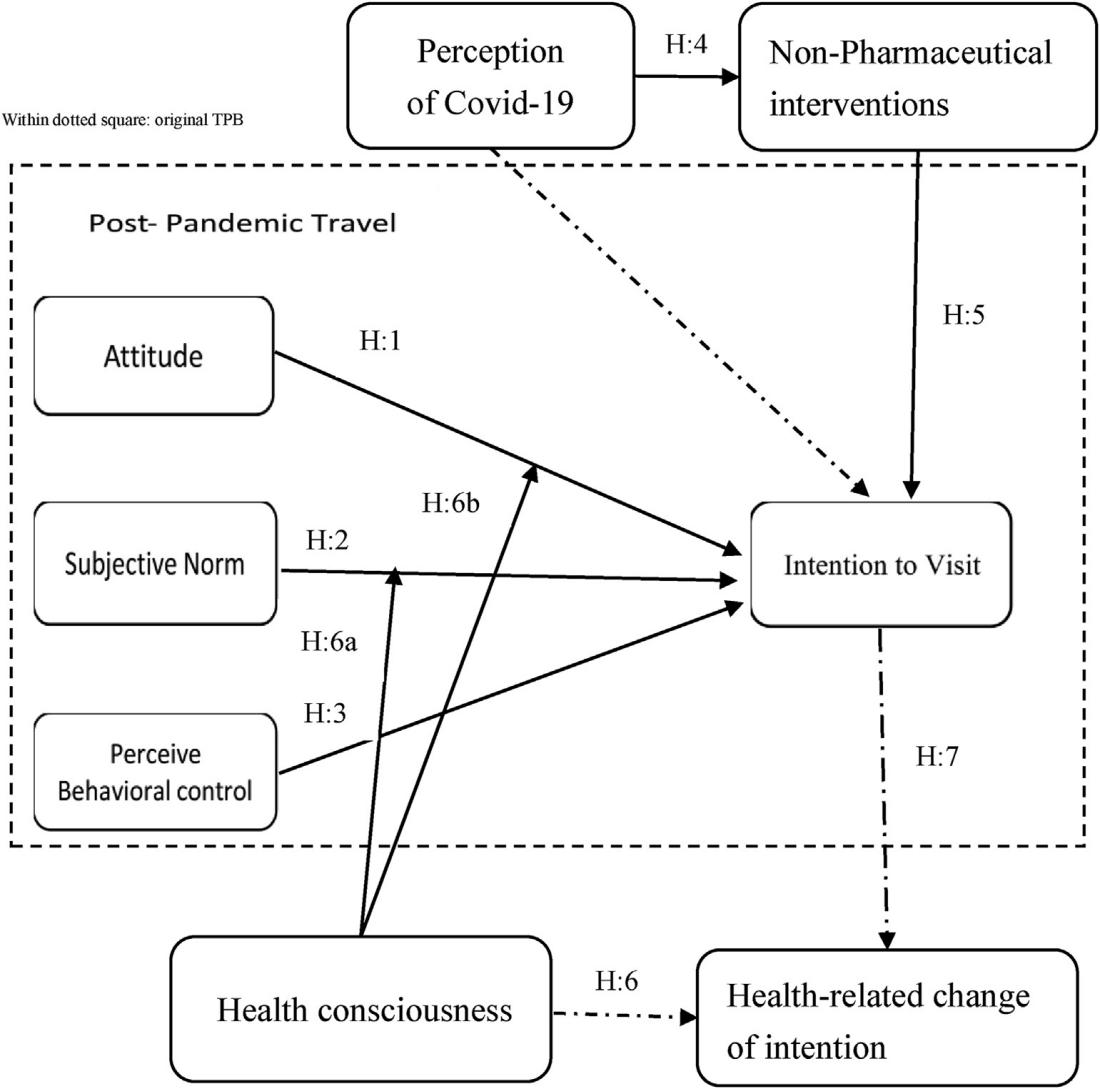
Proposed model.

Some procedures were conducted to reach the aims in this study such as Validity and reliability Index, Discriminant validity, Structure Testing Model, EFA, PLS-SEM, Hypothesis Testing (t-test). Quantitative analysis approach was used in this study to answer the hypothesis proposed model in Figure 1. Institute of Educational Development-West Nusa Tenggara (LPP-NTB) Indonesia that funds this research did not any require ethical approval for this study. The researchers assured that the anonymity of the respondents.

### 3.1 Data collection procedure

Some researchers in tourism and hospitality field used as an online survey for collecting data (Han et al., 2009; Kim and Ok, 2009). This study used as an online survey method to collect potential data. List of questions in the questionnaire was sent to local tourist in Indonesia who want to visit a local destination post-pandemic covid-19. The sample of this study was spread through online and random ways. The internet survey has distributed the questionnaire to potential local tourist chosen randomly. The survey invitation email was delivered to the potential respondent. Online survey (Google form platform) was used in this study for collecting the data.

Respondents in this study were local tourists who will be visit a local destination in Indonesia post-pandemic covid-19. The respondents in this study from several part or provinces of Indonesia as a representative of sampling. The respondents were invited to click the link to fill out the questionnaire. All respondents are entirely guided to check the instructions and read how to fill out the questions. The respondents of this study who have older than 18 years were asked to fill out the questionnaire. The number of questionnaires was sent out to the respondent around 350 respondents while collecting data from September to October 2020. The purposive sampling was used in this study (Cheah et al., 2020) to analyze who want to visit a local destination post pandemic covid-19. The total of 200 useable participants of local tourist for the study were collected through the collection data process. The validating model in this study with small sample size because SEM-PLS particularly used well with small sample size (Hair et al., 2012). Another study related to sample size proposed by (Roscoe, 1975) cited in (Sekaran and Bougie, 2016) said that the minimum sample size of more than 30 and less than 500 is approved for most studies. From this statement, the proposed sample size in this study 200 domestic tourists.

### 3.2 Questionnaire measurement and development

In order to construct this study, some questions were adopted from some sources to arrange the questions. The variables of this study were adopted by TPB constructs Attitude (Li et al., 2020; Wang and Ritchie, 2012), Subjective Norm (Chen and Tung, 2014; Li et al., 2020), Perceive Behavioral Control (Chen and Tung, 2014; Li et al., 2020; Wang and Ritchie, 2012), variable of intention to visit adopted from (Chen and Tung, 2014; Li et al., 2020; Pahrudin et al., 2020), variable perception of covid-19 was developed from (Lee et al., 2012), variable of Non-Pharmaceutical intervention was developed from (Lee et al., 2012), variable of health consciousness/health risk was adopted from (Zeng et al., 2021) and variable Health-related Change intention was adopted from (Juschten et al., 2019).

The questionnaire was designed in bilingual, English and Indonesian language because the respondents in this study are Indonesian tourist who wants to visit a local destination post-pandemic covid-19. The scales of previous studies were adopted in this study. Multiple items were used in this Study with Likert-type scale with seven scales (Scale 1: Strongly Disagree, and Scale 7: Strongly Agree) and for health related to change of intention (Scale 1: Never, and 7: Always).

### 3.3 Data analysis of study

To analyze each variable in this study, we approved Structural Equation Model-Partial Least Square (SEM-PLS) approach to analyze the data with smartPLS software (version 2.0). First, descriptive statistics was used to analyze the characteristic of respondents such as gender, education, background, age, etc with SPSS software tools. The sample size of this study was adequate to analyze data using multivariate data analysis (Structural Equation Model). PLS-SEM is used to look for latent patterns in the data and to know more about how the variables are related (Hair et al., 2014). To evaluate the developed measurement scale according to the three criteria: (1) the composite reliability (CR) of latent variables should not be less than 0.7 (Nunnally and Bernstein, 1994), (2) the average variance extracted (AVE) should not be less than 0.5 (Fornell and Larcker, 1981b) and (3) the factor loading of confirmatory factor analysis (CFA) should not be less than 0.6 (Nunnally and Bernstein, 1994).

## 4 Result and discussion

### 4.1 Sample characteristic

The first questions in the questionnaire are to getting general information about respondents or demographic information. Demographic information includes gender, age, education, income, and occupation. The proportion of gender divided into two genders, male and female. Male and female respondents were 43% and 57%, and most of the respondents were aged 18–24 with 57.7%. Educational respondents background was finished or dominated by senior high school with 41%. Respondents Income (Monthly) dominated by less than Indonesian Rupiah (IDR) 1.000.000 or 58%. Mostly Respondents occupation as a student with 57.5 %. The respondent information was involved with personal information about the respondents. Respondent in this Study was collected from the tourist who wants to visit a local destination post-pandemic (COVID-19). The demographic information about the respondents could be seen in Table 1.

**Table 1:** Demographic respondent profile (N = 200).

Variable	Responses	Frequency	Per cent
Gender	Male	86	43.0
	Female	114	57.0
Age (year)	18–24	115	57.5
	25–34	56	28.0
	35–44	18	9.0
	45–54	10	5.0
	More than 55	1	.5
Education	Senior High School	82	41.0
	Bachelor	68	34.0
	Master	41	20.5
	Doctor	9	4.5
Income Monthly (IDR)	Less than IDR 1.000.000	116	58.0
	IDR 1.000.000–5.000.000	58	29.0
	IDR 5.000.000–10.000.000	18	9.0
	IDR10.000.000–15.000.000	2	1.0
	more than IDR 15.000.000	6	3.0
Occupation	Student	115	57.5
	Full-time job	36	18.0
	Part-time job	18	9.0
	Businessman	6	3.0
	Household keeping	5	2.5
	Professional	20	10.0

### 4.2 Validity and reliability analysis

The process of validity and reliability of this research, internal consistency was used, measured with Composite Reliability (CR) score. The reliability of each item is tested by the three indicators. To determine according to the suggested factor loadings that should be above .50 (Hair et al., 1992). In respect of each potential variables, it has to approach above .70 to composite reliability (Fornell and Larcker, 1981a), above .70 of Cronbach's alpha value (Fornell and Larcker, 1981b) (Fornell and Larcker, 1981b; Nunnally and Bernstein, 1994) and above .50 of Average Variance Extracted (AVE) (Fornell and Larcker, 1981b). In respect of the test of validity, the AVE of each potential variables needs to be greater than .50 to be determined as convergent validity, and the test standard of the square root of AVE is .70 over to be determined as the discriminate validity.

The standard of value of Composite Reliability of this research is .70, and all constructs were higher than .70, which ensure the internal reliability of items in this study. As given in Table 2, the value of Composite Reliability of this research can be seen in Table 2. To ensure the validity of latent variables, we assessed both convergent and discriminant validity. The first, convergent validity was assessed by examining both the average value extracted (AVE) score and loading Factors of each indicator related to constructing. A confirmatory factor analysis was adopted to compute the factors loading. The result shows that the value of AVE ranged from .614 to .912, which are well above .50 of the standard value. The factor loading ranges from .701 to .972; it is mean all variable supporting of convergent validity.

**Table 2:** The construct and item measurement of Factor Loading, AVE, CR and CA.

Construct and Item Measurement	Factor Loading	AVE	CR	Cronbach Alpha	
**Attitude**					
1. After this pandemic covid-19 is over, I believe that it is still a good idea to visit a local destination that I intended visiting.	.882	.722	.886	.809	
2. After this pandemic covid-19 is over, I am glad about going to visit a local destination that I intended visiting originally.	.919				
3. After this pandemic covid-19 is over, I would be positive about going on holiday to visit a destination that I intended on visiting.	.737				
**Subjective Norm**					
1. After this pandemic covid-19 is over, I intend on going on travel a local destination that I have chosen before.	.887	.760	.905	.842	
2. After this pandemic covid-19 is over, my friends and I plan to go to travel a local destination that they had chosen to visit originally.	.864				
3. After this pandemic covid-19 is over, most friends who are closed recommend to me to travel a local destination.	.864				
**Perceive Behavioral Control**					
1. After this pandemic covid-19 is over, I have time and opportunities to visit a local destination that I intended on visiting originally.	.809	.694	.901	.854	
2. After this pandemic covid-19 is over, I have resource ability to go to visit a local destination that I intended visiting.	.767				
3. After this pandemic covid-19 is over, I'm confident that I could visit a local destination that I intended visiting.	.893				
4. After this pandemic covid-19 is over, I'm capable to go to visit a local destination that I have chosen before.	.859				
**Intention to Visit**					
1. After this pandemic covid-19 is over, I will go to travel a local destination in the future.	.949	.912	.969	.952	
2. After this pandemic covid-19 is over, I am excited to visit a local destination in the near future.	.948				
3. After this pandemic covid-19 is over, I am planning to visit a local destination in the future.	.967				
**Perception of covid-19**					
1. Travelling during covid-19 is dangerous.	.701	.620	.890	.845	Remove: I have much information about covid-19.
2. Covid-19 is a very scary disease.	.898				
3. Covid-19 is more dangerous than another pandemic such as SARS and avian flu.	.785				
4. I am afraid of covid-19 disease.	.837				
5. People around me seem to refrain from travelling due to covid-19.	.697				
**Non-Pharmaceutical intervention**					
1. I will check the information about covid-19 by visiting the website of the government before travelling to another destination.	.731	.614	.917	.894	Remove: I will read the and check precautions about covid-19 through the hospital doctors or health center of covid-19 before travelling.
2. I will prepare a first aid kit for covid-19 before travelling to another destination.	.770				
3. I will wash my hands frequently when travelling.	.860				
4. I will restrain and avoid to touching the eyes, nose or mouth while travelling.	.722				
5. I will keep distancing while travelling.	.835				
6. I will frequently use a mask while travelling.	.727				
7. I will restrain and keep distance from meeting people for a while after travelling.	.829				
**Health consciousness/Risk perceive**					
1. I don't think that travelling is damaging for me during pandemic covid-19.	.972	.768	.867	.747	Remove: Health is very important to me than travelling nowadays, and health means a lot to me.
2. I think that travelling is harmless for me in pandemic covid-19 during use health protocol.	.768				
**Health-related Change Intention**					
1. How frequently would you intend to visit a destination in the future destination?	.869	.786	.880	.729	Remove: How often would you consider visiting such a destination in the pandemic period?
2. How much would you like to visit a destination in the pandemic period?	.904				

For this study, the AVE root of the individual potential variable brought up by (Chin, 1998) to test the discriminate validity that should be greater than the common variate relationships of other potential variables of the potential variables and models. In addition, researchers suggest that the test standard of AVE root should be at least equal or greater than .70. In this case, the result value of AVE in this study at least equal with .70 (Venkatesh et al., 2012).

Discriminant validity was assessed by comparing the square root of the AVE for each construct against the inter construct correlation. As shown in Table 3, all the diagonal element, which are the square of root AVE, exceed the inter construct correlations, thereby satisfying the discriminant validity.

**Table 3:** Discriminant validity.

	AVE	CR	CA	1.	2.	3.	4.	5.	6.	7.	8.
1. Attitude	.722	.886	.809	.850							
2. Subjective Norm	.760	.905	.842	.656	.872						
3. Perceive Behavioral Control	.694	.901	.854	.558	.695	.833					
4. Perception of Covid-19	.620	.890	.845	.025	.128	.201	.788				
5. Non-Pharmaceutical Interventions	.614	.917	.894	.168	.365	.311	.571	.784			
6. Health Consciousness	.768	.867	.747	.044	.146	.185	-.086	-.082	.876		
7. Intention to Visit	.912	.969	.952	.587	.689	.781	.200	.382	.169	.955	
8. Health related change of Intention	.786	.880	.729	.282	.299	.268	-.067	.061	.368	.328	.886

Note: AVE: Average Variance Extracted CR = Composite Reliability, CA = Cronbach Alpha. The average variance was extracted from the square average variance extracted.

### 4.3 Structure testing model

To test the Hypothesis, the explained variance (R^2^) of the dependent variable path coefficient (Beta) and their level significant (t-value), which obtained from bootstrapping with resampling (500 resamples) to assess the significance of the hypothesized relationship. Results of the hypothesis testing summarized by Table 4 and the hypothesized in our research model are supported.

**Table 4:** Structure testing model.

	Original Sample	Standard Error	T Statistics	P Statistics
A1 ← 1. Attitude	.882	.022	40.358	.000
A2 ← 1. Attitude	.919	.018	52.540	.000
A3 ← 1. Attitude	.737	.083	8.907	.000
NS1 ← 2. Subjective Norm	.887	.022	40.298	.000
NS2 ← 2. Subjective Norm	.864	.041	21.324	.000
NS3 ← 2. Subjective Norm	.864	.022	39.194	.000
B1 ← 3. Perceive Behavioral Control	.809	.039	20.986	.000
B2 ← 3. Perceive Behavioral Control	.767	.044	17.379	.000
B3 ← 3. Perceive Behavioral Control	.893	.024	37.291	.000
B4 ← 3. Perceive Behavioral Control	.859	.017	49.600	.000
PC1 ← 4. Perception of Covid-19	.701	.054	13.018	.000
PC2 ← 4. Perception of Covid-19	.898	.017	54.155	.000
PC3 ← 4. Perception of Covid-19	.785	.039	20.170	.000
PC5 ← 4. Perception of Covid-19	.837	.032	26.141	.000
PC6 ← 4. Perception of Covid-19	.697	.051	13.648	.000
NPI1 ← 5. Non-Pharmaceutical Interventions	.731	.044	16.494	.000
NPI3 ← 5. Non-Pharmaceutical Interventions	.770	.039	19.700	.000
NPI4 ← 5. Non-Pharmaceutical Interventions	.860	.024	36.494	.000
NPI5 ← 5. Non-Pharmaceutical Interventions	.722	.071	10.182	.000
NPI6 ← 5. Non-Pharmaceutical Interventions	.835	.034	24.303	.000
NPI7 ← 5. Non-Pharmaceutical Interventions	.727	.058	12.473	.000
NPI8 ← 5. Non-Pharmaceutical Interventions	.829	.029	28.763	.000
HC1 ← 6. Health Consciousness	.972	.119	8.190	.000
HC2 ← 6. Health Consciousness	.768	.204	3.762	.000
I1 ← 7. Behavioral Intention to Visit	.949	.012	79.229	.000
I2 ← 7. Behavioral Intention to Visit	.948	.011	87.918	.000
I3 ← 7. Behavioral Intention to Visit	.967	.007	147.707	.000
HI2 ← 8. Health-related change of Intention	.869	.061	14.303	.000
HI3 ← 8. Health-related change of Intention	.904	.037	24.147	.000
A1∗HC1 ← 1. Attitude ∗ 6. Health Consciousness	.924	.019	50.006	.000
A1∗HC2 ← 1. Attitude ∗ 6. Health Consciousness	.853	.044	19.420	.000
A2∗HC1 ← 1. Attitude ∗ 6. Health Consciousness	.927	.019	49.631	.000
A2∗HC2 ← 1. Attitude∗ 6. Health Consciousness	.865	.043	20.065	.000
A3∗HC1 ← 1. Attitude ∗ 6. Health Consciousness	.899	.025	35.445	.000
A3∗HC2 ← 1. Attitude ∗ 6. Health Consciousness	.849	.044	19.263	.000
NS1∗HC1 ← 2. Subjective Norm ∗ 6. Health Consciousness	.914	.017	54.293	.000
NS1∗HC2 ← 2. Subjective Norm ∗ 6. Health Consciousness	.884	.029	30.602	.000
NS2∗HC1 ← 2. Subjective Norm∗ 6. Health Consciousness	.915	.017	55.556	.000
NS2∗HC2 ← 2. Subjective Norm ∗ 6. Health Consciousness	.884	.029	30.638	.000
NS3∗HC1 ← 2. Subjective Norm ∗ 6. Health Consciousness	.921	.015	60.000	.000
NS3∗HC2 ← 2. Subjective Norm ∗ 6. Health Consciousness	.890	.027	32.965	.000

### 4.4 Hypothesis analysis

The measurement model is developed in this study with criteria with validity and reliability in the model of Structural Equation Model-Partial Least Square (SEM-PLS). There are two critical perspectives for testing and analyzing the structural model with the PLS. The first perspective is to standardize the path coefficient; the second is to determine the explanatory model with R (Fornell and Larcker, 1981b; Hair Jr. et al., 2014). Each potential paths coefficient among variables and the result of the R-value reveals the level of goodness fitting of the structural model and the empirical data. The standardized path coefficient has to approach statistical significance at level 5%; R is applied to determine the analytical capability that the higher the R-value is higher the better analytical capability it has.

The Hypothesis result showed that the attitude had a significant impact on behavioral intention to visit (H1: β = .456, t = 3.009). Furthermore, subjective norm had no significant impact on intention to visit (H2: β = .002, t = .990). Thus, this hypothesis was rejected. The variable Perceive Behavioral Control had a significant impact on behavioral intention to visit (H3: β = 527, t = 10.752). The variable perception of Covid-19 had a significant impact on variable non-pharmaceutical intervention (H4: β = .571, t = 10.752). The variable of non-pharmaceutical intervention had a significant impact on variable behavioral intention (H5: β = 132, t = 2.590). The variable health consciousness had no significant impact on variable behavioral intention (H6: β = .542, t = 1.374). Thus, this hypothesis was rejected. The variable behavioral intention to visit had a significant impact on variable health related to change of intention (H7: β = 328, t = 5.330).

In this study, we examined the impact moderator variable to the theoretical framework. The analysis of the moderator variable investigated the framework of how the role the variable health consciousness to the variable attitude and subjective norm. The result showed that the variable health consciousness had the negative relationship between attitude variable and behavioral intention to visit (H6a: β = -.865, t = 2.061) and this Hypothesis had a significant impact. The role moderator variable health consciousness to the variable subjective norm had a positive relationship between subjective norm and intention to visit (H6b: β = .863, t = .389) and the hypothesis was rejected. The description of the analysis of the overall research hypothesis is as Table 5.

**Table 5:** Hypothesis analysis.

Variable	Original Sample (Estimated β)	Standard Error	T Statistics	P Statistics	Status
1. Attitude → 7. Intention to Visit	.456	.152	3.009	.003∗∗∗	Supported
1. Attitude∗ 6. Health Consciousness → 7. Intention to Visit	-.865	.420	2.061	.040∗∗∗	Supported
2. Subjective Norm → 7. Intention to Visit	.002	.175	.012	.990∗	Rejected
2. Subjective Norm∗ 6. Health Consciousness → 7. Intention to Visit	.357	.413	.863	.389∗	Rejected
3. Perceive Behavioral Control → 7. Intention to Visit	.527	.067	7.905	.000∗∗∗	Supported
4. Perception of Covid-19 → 5. Non-Pharmaceutical Interventions	.571	.053	10.752	.000∗∗∗	Supported
5. Non-Pharmaceutical Interventions → 7. Intention to Visit	.132	.051	2.590	.010∗∗∗	Supported
6. Health Consciousness → 7. Intention to Visit	.542	.395	1.374	.170∗	Rejected
7. Intention to Visit → 8. Health-related change of Intention	.328	.062	5.330	.000∗∗∗	Supported

∗∗∗Significant at the p < 0.05 level (two-tailed).

A path coefficient represents the strength and orientation of the relationships among the research variables; the test of a path coefficient should show the significance and be accordant with the predicted orientation in the research Hypothesis to establish the relationships among the predictive and validity index variables. The Smart PLS 2.0 is adopted in the study to proceed to test the structural model; the Structural Equation Modelling (SEM) (path analysis) and the results are presented; the underlined values are the standardized regression coefficients (β values).

### 4.5 Explanatory capability

The R values represent the predictive capability of the research model, which is the percentage of the variance of the outer variables can explain the inner variables. In respect of the analysis of causal relationships (of constructed model), it depends on whether if the coefficient of the standardized route approaches statistical significance, and the explanatory capacity of the R square determination model (Fornell and Larcker, 1981b; Hair Jr. et al., 2014). Table 6 provided the result of explanatory capability.

**Table 6:** Explanatory capability.

	AVE	CR	R^2^	CA	Communality	Redundancy	GOF
1. Attitude	.722	.886		.809	.722		.529
1. Attitude ∗ 6. Health Consciousness	.786	.957		.947	.786		
2. Norm	.760	.905		.842	.760		
2. Norm ∗ 6. Health Consciousness	.813	.963		.954	.813		
3. Perceive Behavioral	.694	.901		.854	.694		
4. Perception of Covid-19	.620	.890		.845	.620		
5. Non-Pharmaceutical Interventions	.614	.917	.326	.894	.614	.197	
6. Health Consciousness	.768	.867		.747	.768		
7. Intention to Visit	.912	.969	.691	.952	.912	.299	
8. Health related change of Intention	.786	.880	.108	.729	.786	.084	

The Goodness of Fit index is a single measurement of the performance model and the structural model in this study. The goodness of Fit (GoF) value is obtained from the square root of the average community index multiplied by the average value of the R^2^. The goodness of Fit Value is from 0-1 with interpretations of value: .1 (Small Goodness of Fit), .25 (Moderate Goodness of Fit), and .36 (Large Goodness of Fit). From the table, the value of Goodness of Fit obtained .529 or 52.9% the variable of attitude, subjective norm and perceive behavior control, and intention are explained the structure model. We can conclude that the Goodness of Fit in this Study is large because of the value of GOF more than .360. From the result, we conclude that the value of R square variable of intention to visit .691. The variable Non-Pharmaceutical Intervention (NPI) to explain the model by .326 and variable health related to change the intention by .108 to explain the model of this study.

### 4.6 Discussion

Under the situation and condition of pandemic covid-19 in the world that given huge impact in all sectors such as economic, social, health, tourism and etc. tourism sector has been faced difficult situation due to covid-19 and require the fast respond and adaptation new strategy to achieve new normal. Knowledge is limited about the pandemic covid-19 impacted in tourist intention to visit a destination post-pandemic. Our theoretical framework tried to build a framework in tourism sector related to theory planned behavioral (TPB) and health consciousness, perception of covid-19 and non-pharmaceutical intervention on tourist to visit a destination post-pandemic covid-19 in Indonesian context.

The results showed that the decision of tourist to visit a local destination post-pandemic covid-19 based on theory planned behavior are generally significant. Variable attitude had a positive relationship and significant to variable intention to visit a destination post-pandemic covid-19. Furthermore, the variable subjective norm in this study had not significant to variable intention to visit a destination, and the Hypothesis was rejected. The last variable in TPB is perceived behavioral control. Perceive Behavioral Control had a significant impact and positive relationship toward variable intention to visit a local destination post-covid-19. The Theory of Planned Behavioral (Attitude, Subjective Norm and Perceive Behavioral control) has been tested in the tourism sector, and behavior studies and the result showed that had a positive relationship (Hwang et al., 2020; Kim and Hwang, 2020; Wu et al., 2017a, Wu et al., 2017b). The other studies, PBC was a usually strong predictor of intention on in context TPB studies (Armitage and Conner, 2001; Davis et al., 2002; Lam and Hsu, 2004). The study from (Ajzen, 1991) said that the concept of TPB, in which attitude toward the action has a positive impact, perceive social pressure and perceive ability toward action have a positive impact to customer behavioral intention of customers.

In addition, the path coefficient variable subjective norm had not significant through variable intention to visit with the value (*p > 0.05*). The variable subjective norm had insignificant to variable intention to visit a local destination post-pandemic because the tourist did not have the plan to visit a destination post-pandemic because spread illness covid-19 and tourist try to avoid it. In the context of Indonesian tourists, the subjective norm as an essential factor to make a decision to visit a local destination due to pandemic covid-19. Indonesian tourist felt fear and avoid the spread of illness covid-19 during a pandemic because of some destination regions unstable yet to be a safe destination in Indonesia due to did not found covid-19 vaccine yet.

The variable perception of covid-19 had a significant impact on non-pharmaceutical intervention in post-pandemic covid-19. It can conclude that tourist cares about their health because there was a significant influence between perception of covid-19 and non-pharmaceutical intervention toward intention to visit a destination. Consumers during an outbreak non-pharmaceutical intervention is the main way in reducing the risk of spread illness while visiting a destination (Lee et al., 2012). The consumers showed that some factors such as intention, perceive behavioral control, previous behavior, and non-pharmaceutical intervention are the main role for traveler intention. Therefore, it is important to Hypothesis the impact of perception of covid-19 and the non-pharmaceutical intervention toward an intention to visit a destination. The results implied that the perception of covid-19 contributed to the tourist behavior and self-preventive when their intention to visit a local destination post-covid 19 with safer tourism destination Indonesia.

The result reveals that the perception of covid-19 had significantly influenced intention to visit a destination. This finding indicated that the perception of covid-19 positively impacted the intention to visit a destination, this result in line with the previous studies demonstrated the relations between perception of disease, attitude and intention (Reisinger and Mavondo, 2005a; Sönmez and Graefe, 1998). It can be concluded that NPI had the main role in avoiding the spread of illness where the study showed that there are significantly impacted among perception of covid-19, NPI and Intention to visit, this is related with (Lee et al., 2012), who said that pandemic H1N1 2009 significantly impacted in intention to international visit toward non-pharmaceutical intervention. Where the study indicated that the there is significant relation between perception of covid-19 and therefore the tourist beliefs through perception of covid-19 as a risk and non-pharmaceutical intervention. This result in line with (Lee et al., 2012) that the perception of the 2009 H1N1 significantly impacted international visit intention through non-pharmaceutical intervention. In hence, this study also supported by (Lee et al., 2012) said that to reduces infection risk and emphases intention, non-pharmaceutical intervention is the main role for personal prevention.

According to the theory of self-consciousness, attitude and behavior are elements that can predict the self-consciousness, which expand to health consciousness and health behavior. The result showed that during illness covid-19, tourists health consciousness had a significant impact as a moderator variable through attitude and intention to visit a destination. This finding means that during and post-pandemic covid-19, the tourists had attitude and information about health consciousness to visit intention post-pandemic covid-19 in local destination of Indonesian. Health consciousness can influence the attitude and intention because the person or tourists know and aware about the health during pandemic. Health consciousness is part of the knowledge to make decision in their trip during pandemic and it is part of the risk perceive. It is relevant with some studies in health consciousness and health behavior in decision to buy healthy food and looking for medical service (Justina et al., 2017; Mai and Hoffmann, 2017). However, as a moderator variable, health consciousness had not significant impact through the subject norm and intention to visit. It is notable that variable subjective norm of tourist had intention to visit a destination post-pandemic with considering health consciousness. This finding indicate that tourist believes that would be had intention to visit a destination post-pandemic although in the future situation and condition under controlling such as find a vaccine, use health protocol, physical distancing and etc.

Health consciousness is the main role during a pandemic because the people could be avoided the spread of illness or spread the virus with health behavior. In this Study, Variable health consciousness toward variable intention to visit had no significant impact or the Hypothesis was rejected. This result implied that lack of tourist health consciousness during traveling in intention to visit in post-pandemic covid-19 would be giving impact to tourist in infected by covid-19 or illness because of the health consciousness is less. In addition, variable intention to visit had significantly impact toward health related to change of intention. The result means that the tourist cares about the health in making the decision in intention to visit post-pandemic. Based on the risk theory, tourists tried to avoid negative experiences and always seeking to maximize their satisfaction. The other word, that high risk perceive gives impact on behavior lower buying (Lim, 2003).

Many researches focused on tourist travel intention and directly impacted in risk perceived (Al-Ansi et al., 2019; Olya & Al-ansi, 2018; Reisinger and Mavondo, 2005b). in marketing study, the risk was found by (Bauer, 1960), and said that consumer behavior contains both risk and uncertainty caused by their action and some unpleasant. On the other hand, if a global pandemic occurs, the behaviour of tourists in the tourism sector is influenced by several main factors such as home income, perceptions of health risks, and consumption measures that are reformed due to pandemic constraint (Lee and Chen, 2011) in the tourism sector, all the factors indicated are important in increasing the tourist behaviour related to intention to visit a destination. Morever, the covid-19 disaster is a significant part of the risk, especially are parts of perceived risk, especially health risk, and the result between the perception of pandemic risk and tourist confident. The perception covid-19 changed tourist travel behaviour to visit a destination during pandemic. Tourist’ tried to avoid the risk during the outbreak that can be perceived the risk from the covid-19.

The spread of covid-19 in the world including Indonesia has had big impact on local and international tourism. The pandemic covid-19 has changed the policy to visit a destination in Indonesia with closed border and lockdown causing decrease in international and domestic tourism and sharply declined the economy activity. Several sectors such as tourism and hospitality industry such restaurant, hotel, transportation industry and small medium enterprises have been affected by covid-19 in Indonesia. Closing the economy activity that has been impacted in several sectors such as tourism facilities, hotel, restaurants, and tour operations resulted in lost income and forced the tourism sector leading the unemployment. Tourism sector in Indonesia has created 1.4 million their loss jobs in formal sectors (hotels, restaurants, and tour operators) and 314.833 people losing employment in the informal sector (Cahyadi and Newsome, 2021). In indonesia since april 2020 the destinations and tourist attraction are closed due to covid-19.

Several sectors such as tourism and hospitality industry such restaurant, hotel, transportation industry and small medium enterprises have been affected by covid-19 in Indonesia. The passengers arrived on international route in Indonesia have decreased from 1,5 million on December 2019, down 450 thousand to 1.15 million in January 2020. Compare to January 2019, it is lower 15% (Weible et al., 2020). In addition, in the beginning of covid-19 in Indonesia 735 international flights were cancelled to Indonesia (Gandasari and Dwidienawati, 2020) and tourism sector in Indonesia suffered a loss around 1.5 billion US dollar. The impact of tourism sector declined due to covid-19 has been affected in small and medium enterprises in micro food and beverage reached 27%. Therefore, the impact of covid-19 on tourism sector decreased from 6.29 million people in January and became 5.79 million in February 2020 or decreased by 8.08% in domestic flights (Dwyer et al., 2020).

Since the situation during covid-19, the Indonesian government regulated the Large-Scale Social Restriction (PSBB). The regulation under the Indonesian's government policy try to restrict the people from gathering a crowd. However, the policy of PSBB is partially regulated only in the regions where the covid-19 cases are many as well as in the Java island region. Thus, several tourism destinations in Indonesia are decreased by implying the regulations in PSBB.

The government took the various regulations in the context in handling a pandemic such as the regional quarantine policy or lockdown, a large-scale social restriction (PSBB) and local scale community activities restriction to avoid the spread of disease. However, there has been a concern that such restrictive policies would hit the tourism sector, especially the regions whose regional revenues are highly dependent on the tourism sector. Indeed, tourism-related businesses have been closed down and did not earn enough revenues resulting in terminations of their employees and creating unemployment. Likewise, the slowing down of tourism in those regions has also affected the regional economy in general. In Bali alone, for example, the Covid-19 Pandemic has caused economic losses up to USD 9.7 trillion per month which has a domino effect on other economic sectors in Bali (Rosidin, 2020).

Due to the covid-19, tourist behavior also was changed. The tourist will be considering the destinations safer and heathy life while traveling and changing the behavior during the travelling post pandemic covid-19. The present study finds to explain the tourist behavior to visit a local destination post pandemic covid-19 in Indonesia using theory planned behavior and non-pharmaceutical intervention and health consciousness and perception of covid-19. It indicates that tourists’ behavior post covid-19 outbreak in Indonesia influenced by some factors such attitude, perceive behavior control, non-pharmaceutical intervention to visit a local destination post pandemic covid-19. These findings can also help the authorities in tourism sector in Indonesia to find best way to avoid the spread of virus covid-19 during traveling to local destination. Therefore, these finding can be also used to inform the policymakers including government and public health institution in Indonesia to design the health protocol intervention related to the tourist who will to visit a local destination post pandemic covid-19 such as wear mask, using hand sanitizer, social distancing and healthy life during traveling to the local destination.

The new findings in this research indicated that the important of health protocols during pandemic covid-19 and post pandemic covid-19 can change the Theory of Planned Behavior constructs to give impact on behavior of tourist to visit a local destination in Indonesia. The authors can understand that the influence of tourist awareness to reach leisure during pandemic or post pandemic covid-19 should be address to health protocols. The facts in tourism destination in Indonesia reveals that the tourism destination is under control to visit by the tourist because of using health protocols such as wear a mask, using hand sanitizer, physical distancing and healthy life. This finding implied that tourists can visit a local destination in Indonesia to use health protocols and health awareness to protect himself from the disease during pandemic covid-19 or post covid-19.

Our results in this study gave our insight and understanding about risk in disaster, including disaster in pandemic and perception of crises management. Hence, based on the enhanced impact pandemic covid-19, our study explained how the tourism industry and leisure was changed by the pandemic. The results in this study were given insight into the literature in tourism especially the destination and health perspective related to pandemic or illness. By linking the tourists’ perception of the behavior in pandemic era and their future intention to visit a local destination, our study serves original framework and contribution in tourism sector. The results in this study could be added in knowledge to the literature in tourism, hospitality and marketing.

Being aware about the contribution of TPB constructs such as attitude and perceive behavior control toward intention, non-pharmaceutical intervention toward to tourist visit a local destination, Indonesian government should be deal to maximize the result of TPB constructs and the awareness of tourist about non pharmaceutical intervention to do health protocol to make decision to visit a local destination post pandemic covid-19 for their trips. Another finding from this paper that tourist health consciousness is still low because the result was rejected. These finding are similar with the condition of the number of the cases covid-19 in Indonesia increased. Based on the statistic that the number cases of covid-19 in Indonesia is more than 2 million (The Indonesian COVID-19 Task Force, 2021). During and post-pandemic covid-19 the Indonesian government or policymakers in Indonesia can open the destination with considering the first, involving the community to educate the tourists behavior to do new life and use health protocol based on concept CHSE (Cleanliness, Healthy, Safety and Environmental sustainability). Second, the policy or the law about the local destination should be enter by the travelers for those really safety and healthy to reduce the spread the illness in the destination. On the other hand, the local destination and attraction providers together to promote health protocol during traveling a local destination post pandemic by providing easily accessible hygiene to the tourists.

## 5 Conclusion, limitations and implications

This study focused on tourists’ behavior and intention to visit a destination in Indonesia post pandemic covid-19. The theory of planned behavior (TPB) construct had influenced the intention to visit a destination related to perception of covid-19, non-pharmaceutical intervention and health consciousness while taking a visit post-pandemic-19. Beyond the study, it is important to all practitioners, tourist marketers, leisure industries, government and stakeholders might read this paper for recognition of the behavior and intention to visit a destination post-pandemic covid-19 to prepare well.

During pandemic covid-19 situation, tourism sector is one of the sectors with uncertainty. This study shows that local tourists will be always use non-pharmaceutical intervention and health protocol such as keep social distancing, wear mask, healthy life during travelling to the local destination in sort term and middle term in the future because the situation under covid-19 pandemic is uncertainty. Travelling for travelers in the future under anxiety because of the new variants (variant alpha, delta, gamma) covid-19 have found in Africa, South America and South Asia. The researchers believe that what we have proposed research model in this research can give a new contribution in developing tourism sector empirically during and post pandemic covid-19 in Indonesia. This research can give contribution to the government to protect the tourist from the disease covid-19 with applying the health protocol during and post pandemic covid-19. This finding also can contribute to intervene in the Theory of Planned Behavior (TPB) and giving an impact on the behavior of tourists to visit a local destination post-pandemic covid-19.

Our study has limitations and gives a chance to explore more for further research. The study examined the TPB in tourism sectors, especially in the destination. The study approach to examine the TPB concept related to post-pandemic covid-19 and linked to perception of covid-19, Non-Pharmaceutical Intervention and health consciousness of tourist to make a decision to visit a local destination in Indonesia. In addition, our study focused on tourist intention to visit a local destination in Indonesia, and a new study could be conducted in international tourists in different countries. This could be investigated and conducted by other researchers to fill out the research gap.

Due to an urgent of the topic and time limitations of research, we collected the data from an online questionnaire to avoid the spread of illness covid-19 and using a quantitative approach to analyze the data. Hence, the next researchers could be using the different approach to assess the model such as, interview approach, focus group discussion and getting more sample. In addition, the study might be conducted in developed and developing countries because of the different tourist behavior.

The main theoretical implications in this study are follows. First, this study provided for applicability of the TPB framework to visit intention a destination in post pandemic under the public health emergencies. Even though, the concept of TPB has been applied in several previous studies on visit intention a destination, few studies have discussed the TPB framework in context of public health emergencies. This study extended the TPB framework in tourism research related to post pandemic covid-19. Second, this study provided the basic understanding for explaining tourist's visit intention during the pandemic and post pandemic disease, because the extended TPB framework has superiority ability to explain greater the intention than the original TPB model. This study can be an understand for the researchers to study tourists' intention post pandemic. Third, this study found that the perception of covid-19 toward non-pharmaceutical intervention affect their intention to visit post pandemic. The results in this study are important in theoretical extensions of research which offering the insight for tourists' visit intention a destination post pandemic.

The finding of this study also offers several implications to the government, agencies, traveler marketers, hospitality industries, and stakeholders. The first, tourist visit intention to destination is resilient during pandemic covid-19 because the potential of tourist perceives non-pharmaceutical intervention such as washing hand, wear a mask while travelling, keep social distancing, and checking the information about the covid-19 as the ways to avoid the spread of illness during a taking journey. Guideline for non-pharmaceutical intervention hygiene information in public destination and safety information such as airline onboard publications, online information, it will be easy for the tourist to aware about their health consciousness. The second, tourist operators and the government should establish information about the covid-19, campaign about NPI and health consciousness during pandemic covid-19 while taking a trip for the tourist, passengers or guests. These are the complexity the problem in the tourism industry to face a during and post pandemic covid-19 in global air transport, and practical issues in tourism-related to non-pharmaceutical intervention and health consciousness that give impact to tourist intention to visit.

## Declarations

### Author contribution statement

Li-Wei Liu: Performed the experiments; Analyzed and interpreted the data; Wrote the paper.

Pahrudin Pahrudin: Conceived and designed the experiments; Performed the experiments; Wrote the paper.

Chien-Ting Chen: Conceived and designed the experiments; Contributed reagents, materials, analysis tools or data; Wrote the paper.

### Funding statement

This work was supported by Institute of Educational Development-West Nusa Tenggara (LPP-NTB Indonesia).

### Data availability statement

Data included in article/supplementary material/referenced in article.

### Declaration of interests statement

The authors declare no conflict of interest.

### Additional information

No additional information is available for this paper.

## Appendix A Supplementary data

Research_Questionnaire Research_Questionnaire
