# Supplementary material for: A modified theory of planned behavioral: A case of tourist intention to visit a destination post pandemic Covid-19 in Indonesia
Source: Heliyon. 2021 Oct 21;7(10):e08230. doi: 10.1016/j.heliyon.2021.e08230 (PMC8529901; doi:10.1016/j.heliyon.2021.e08230)
Supplement: Research_Questionnaire [file mmc1.docx]

**The Questionnaire of Research**

Demographic information

**Gender :**

1. Male
2. Female

**Age :**

1. 18-24
2. 25-34
3. 35-44
4. 45-54
5. More than 55

**Education :**

1. Senior high school
2. Bachelor
3. Master
4. Doctor

**Income :**

1. Less than IDR 1.000.000
2. IDR 5.000.000-10.000.000
3. IDR 10.000.000-15.000.000
4. More than IDR 15.000.000

**Occupation :**

1. Student
2. Full-time job
3. Part time job
4. Businessman
5. Household keeping
6. Professional

**Research Questions.**

| **Variables** | **Scale** | | | | | | |
| --- | --- | --- | --- | --- | --- | --- | --- |
| **Attitude** | **1** | **2** | **3** | **4** | **5** | **6** | **7** |
| 1. After this pandemic covid-19 is over, I believe that it is still a good idea to visit a local destination that I intended visiting. |  |  |  |  |  |  |  |
| 1. After this pandemic covid-19 is over, I am glad about going to visit a local destination that I intended visiting originally. |  |  |  |  |  |  |  |
| 1. After this pandemic covid-19 is over, I would be positive about going on holiday to visit a destination that I intended on visiting |  |  |  |  |  |  |  |
| **Subjective Norm** |  |  |  |  |  |  |  |
| 1. After this pandemic covid-19 is over, I intend on going on travel a local destination that I have chosen before. |  |  |  |  |  |  |  |
| 1. After this pandemic covid-19 is over, my friends and I plan to go to travel a local destination that they had chosen to visit originally. |  |  |  |  |  |  |  |
| 1. After this pandemic covid-19 is over, most friends who are closed recommend to me to travel a local destination. |  |  |  |  |  |  |  |
| **Perceive Behavioral Control** |  |  |  |  |  |  |  |
| 1. After this pandemic covid-19 is over, I have time and opportunities to visit a local destination that I intended on visiting originally. |  |  |  |  |  |  |  |
| 1. After this pandemic covid-19 is over, I have resources ability to go to visit a local destination that I intended visiting. |  |  |  |  |  |  |  |
| 1. After this pandemic covid-19 is over, I’m confident that I could visit a local destination that I intended visiting |  |  |  |  |  |  |  |
| 1. After this pandemic covid-19 is over, I’m capable to go to visit a local destination that I have chosen before. |  |  |  |  |  |  |  |
| **Intention to Visit** |  |  |  |  |  |  |  |
| 1. After this pandemic covid-19 is over, I will go to travel a local destination in the future. |  |  |  |  |  |  |  |
| 1. After this pandemic covid-19 is over, I am excited to visit a local destination in the near future. |  |  |  |  |  |  |  |
| 1. After this pandemic covid-19 is over, I am planning to visit a local destination in the future. |  |  |  |  |  |  |  |
| **Perception of Covid-19** |  |  |  |  |  |  |  |
| 1. Travelling during covid-19 is dangerous. |  |  |  |  |  |  |  |
| 1. Covid-19 is a very scary disease. |  |  |  |  |  |  |  |
| 1. Covid-19 is more dangerous than another pandemic such as SARS and avian flu. |  |  |  |  |  |  |  |
| 1. I am afraid of covid-19 disease. |  |  |  |  |  |  |  |
| 1. People around me seem to refrain from travelling due to covid-19. |  |  |  |  |  |  |  |
| 1. I have much information about covid-19. |  |  |  |  |  |  |  |
| **Non-pharmaceutical intervention** |  |  |  |  |  |  |  |
| 1. I will check the information about covid-19 by visiting the website of the government before travelling to another destination. |  |  |  |  |  |  |  |
| 1. I will prepare a first aid kit for covid-19 before travelling to another destination |  |  |  |  |  |  |  |
| 1. I will wash my hands frequently when travelling. |  |  |  |  |  |  |  |
| 1. I will restrain and avoid to touching the eyes, nose or mouth while travelling. |  |  |  |  |  |  |  |
| 1. I will keep distancing while travelling. |  |  |  |  |  |  |  |
| 1. I will frequently use a mask while travelling. |  |  |  |  |  |  |  |
| 1. I will restrain and keep distance from meeting people for a while after travelling. |  |  |  |  |  |  |  |
| 1. I will read the and check precautions about covid-19 through the hospital doctors or health center of covid-19 before travelling. |  |  |  |  |  |  |  |
| **Health Consciousness/Risk Perceive** |  |  |  |  |  |  |  |
| 1. I don't think that travelling is damaging for me during pandemic covid-19. |  |  |  |  |  |  |  |
| 1. I think that travelling is harmless for me in pandemic covid-19 during use health protocol. |  |  |  |  |  |  |  |
| 1. Health is very important to me than travelling nowadays, and health means a lot to me. |  |  |  |  |  |  |  |
| **Health-Related Change Intention** |  |  |  |  |  |  |  |
| 1. How frequently would you intend to visit a destination in the future destination? |  |  |  |  |  |  |  |
| 1. How much would you like to visit a destination in the pandemic period? |  |  |  |  |  |  |  |
| 1. How often would you consider visiting such a destination in the pandemic period? |  |  |  |  |  |  |  |
